# Incidence and Outcomes of Advanced Heart Failure in Adults With Congenital Heart Disease

**DOI:** 10.1161/CIRCHEARTFAILURE.122.009675

**Published:** 2022-10-04

**Authors:** Alexander C. Egbe, William R. Miranda, C. Charles Jain, Crystal R. Bonnichsen, Jason H. Anderson, Joseph A. Dearani, Carole A. Warnes, Juan Crestanello, Heidi M. Connolly

**Affiliations:** Department of Cardiovascular Medicine (A.C.E., W.R.M., C.C.J., C.R.B., J.H.A., C.A.W., H.M.C.), Mayo Clinic Rochester, MN.; Department of Cardiovascular Surgery (J.A.D., J.C.), Mayo Clinic Rochester, MN.

**Keywords:** adults, heart disease, heart failure, incidence, mortality

## Abstract

There are limited data about the stage D heart failure (advanced HF) in adults with congenital heart disease. Our study objectives were (1) to determine the incidence of new-onset advanced HF in patients and the relationship between advanced HF and all-cause mortality and (2) to determine the relationship between therapies for advanced HF and all-cause mortality.

**Methods:**

Retrospective cohort study of adults with congenital heart disease at Mayo Clinic (2003–2019). We defined advanced HF using the European Society of Cardiology diagnostic criteria for advanced HF. Therapies received by the patients with advanced HF were classified into 3 mutually exclusive groups (treatment pathways): (1) conventional cardiac intervention, (2) transplant listing, and (3) palliative care.

**Results:**

Of 5309 patients without advanced HF at baseline assessment, 432 (8%) developed advanced HF during follow-up (1.1%/y), and the incidence of advanced HF was higher in patients with severe or complex congenital heart disease. Onset of advanced HF was associated with 6-fold increase in the risk of mortality. Conventional cardiac intervention was associated with significantly higher risk of mortality as compared to transplant listing. The longer the interval from the initial onset of advanced HF to transplant evaluation, the lower the odds of being listed for transplant.

**Conclusions:**

Based on these data, we postulate that early identification of patients with advanced HF, and a timely referral for transplant evaluation (instead of conventional cardiac intervention) may offer the best chance of survival for these critically ill patients. Further studies are required to validate this postulation.

**What is New?**

- The incidence of advanced heart failure (HF) in adults with congenital heart disease was 1.1% per year).
- The onset of advanced heart failure was associated with 6-fold increase in the risk of mortality.
- Conventional cardiac intervention was associated with significantly higher risk of mortality as compared to transplant listing.
- The longer the interval from the initial onset of advanced heart failure to transplant evaluation, the lower the odds of being listed for transplant.

**What are the Clinical Implications?**

- Early identification of patients with advanced heart failure, and a timely referral for transplant evaluation may offer the best chance of survival in this population.

**See Editorial by Cedars and Menachem**

Congenital heart disease (CHD) is the leading cause of cardiovascular death in young adults, and more than half of the patients die from end-stage heart failure (HF).^1–4^ The term HF is used to describe a spectrum of cardiac dysfunction of varying severity ranging from asymptomatic patients (stage A and B HF), symptomatic patients (stage C HF), to severely symptomatic patients that are unresponsive the therapy (stage D HF) based on the classification scheme proposed by the American College of Cardiology/American Heart Association.^5^ Stage D HF (also known as advanced HF) is characterized by persistent and severe symptoms of HF despite optimized medical, surgical, and device therapy.^5^ Patients with advanced HF may be eligible for cardiac replacement therapies, such as heart transplant and mechanical circulatory support to improve quality of life and survival.^5^

A recent study conducted in patients with acquired heart disease showed that advanced HF was present in 42% of patients with HF and that the onset of advanced HF was associated with recurrent hospitalization and death, typically within 1 to 2 years from the onset of advanced HF.^6^ Such data about the prevalence and prognostic implications of advanced HF are lacking in the CHD population. This is because the studies describing the outcomes of HF in adults with CHD, often combined both stage C and stage D HF (advanced HF).^1–4,7,8^ Considering the fact that HF in adults with CHD is the leading cause of cardiovascular mortality in young adults, there is a critical need to define the characteristics and outcomes of this subset of patients, as an important initial step toward designing and implementing treatment strategies to improve outcomes in this population.

The purpose of this study was to determine the incidence and outcomes of advanced HF in adults with CHD. The study objectives were (1) to determine the incidence of advanced HF in patients without advanced HF at baseline, and the relationship between onset of advanced HF and all-cause mortality and (2) to determine the relationship between therapies for advanced HF and all-cause mortality.

## Methods

### Study Population

All data and supporting materials have been provided with the published article. The Mayo Clinic Institutional Review Board approved the study and waived informed consent for patients that provided research authorization. We reviewed the MACHD (Mayo Adult Congenital Heart Disease) Registry and identified adults (≥18 years of age) with CHD that received care at Mayo Clinic from January 1, 2003 to December 31, 2019. The congenital heart lesions were classified as severe CHD (tetralogy of Fallot, truncus arteriosus, pulmonary atresia with intact ventricular septum, transposition of great arteries, univentricular heart, and atrioventricular canal defect) versus nonsevere CHD using the modified CHD severity classification scheme proposed by Marelli et al.^9^ The patients were also classified based on anatomic/physiological subgroups: (1) right-sided lesions (tetralogy of Fallot, Ebstein anomaly, pulmonic stenosis, pulmonary atresia with intact ventricular septum, double-chambered right ventricle, and truncus arteriosus); (2) left-sided lesions (coarctation aorta, aortic stenosis, subaortic stenosis, and Shone complex); (3) systemic right ventricle (congenitally corrected transposition of great arteries and complete transposition of great arteries status post atrial switch operation); (4) Fontan palliation/unrepaired single ventricle; and (5) miscellaneous (this includes the patients that were no classified into these anatomic/physiological subgroups).

### Diagnostic Criteria of Advanced HF

We defined advanced HF using the European Society of Cardiology diagnostic criteria for advanced HF (modified for the CHD population), and the 4 criteria are (1) episodes of congestion, low output, or malignant arrhythmias; (2) evidence of severe cardiac dysfunction, including systemic ventricular systolic dysfunction, nonsystemic ventricular systolic dysfunction, and systemic ventricular diastolic dysfunction; (3) severe exercise impairment; and (4) severe and persistent symptoms of HF (Table S1).^10^ To have advanced HF, a patient must meet all 4 criteria, despite attempts to optimize medical, surgical, and device therapy.

The first clinic visit within the study period was considered as the baseline evaluation. All clinical assessments and cardiac tests performed within 12 months from the baseline evaluation were reviewed in all patients (n=5321) to determine whether advanced HF was present (or not) at the time of baseline evaluation. The patients that met the diagnostic criteria for advanced HF at the time of baseline assessment were considered as having prevalent advanced HF (n=12). These patients were excluded from all subsequent analyses.

For the patients without advanced HF at baseline evaluation (n=5309), we reviewed the medical records and identified patients with 1 or more hospitalizations or emergency department visits for HF or ventricular arrhythmias from the baseline assessment until death or last follow-up through December 31, 2019. Each hospitalization or visit to emergency department was treated as a potential advanced HF index date.^6^ Next, each potential advanced HF index date (including cardiac tests performed within 12 months) was reviewed to determine if the patient met the other advanced HF criteria. The patients that met these criteria were considered as having incident (new-onset) advanced HF.

### Treatment and Outcomes

Among the patients that developed new-onset advanced HF, the types of therapies received were classified into 3 mutually exclusive groups (treatment pathways): (1) conventional cardiac intervention (CCI) defined as transcatheter interventions or cardiac surgical interventions other than heart transplant, (2) transplant listing, and (3) palliative care defined as patients that were evaluated for CCI or transplant but deemed not to be suitable candidates for these therapies. All-cause mortality was ascertained by review of electronic health records and the Accurint mortality database.

### Statistical Analysis

Data were presented as mean±SD, median (interquartile range), and count (%). Between-group comparisons were performed using unpaired *t* test, Wilcoxon rank-sum test, and χ^2^ test. New-onset advanced HF was assessed as a time-to-event outcome using the Kaplan-Meier method, stratified by CHD severity and anatomic/physiological classification, and compared using log-rank test. The relationship between advanced HF and all-cause mortality was assessed using multivariable Cox regression analysis, using the baseline evaluation as time zero, and the onset of advanced HF was modeled as time-dependent covariate. The Cox model was adjusted for age, sex, CHD severity (modeled as categorical variable with the nonsevere CHD group as the reference group), CHD lesion subgroups (modeled as categorical variable with the right-sided lesion as the reference group), comorbidities, global hepatic function (model for end-stage liver disease excluding international normalized ratio score), and systemic ventricular/valvular dysfunction. These covariates were chosen a priori based on known association with clinical outcomes and included in the model using stepwise backward selection with a *P*<0.05 as the criteria for a covariate to remain in the model. The associations between covariates and outcomes were expressed using hazard ratio and 95% CI.

In the subset of patients that developed new-onset advanced HF, the relationship between treatment pathway (modeled as categorical variable using transplant listing as the reference group), and all-cause mortality was assessed using multivariable Cox regression analysis, using the time of advanced HF diagnosis as time zero. Logistic regression analysis was used to determine whether the interval from the initial diagnosis of advanced HF and initial presentation to the transplant clinic was associated with subsequent transplant listing and expressed as odds ratio and 95% CI. These models were adjusted for age, sex, CHD severity, CHD lesion subgroups, and comorbidities, using criteria for section of covariates as described above.

All statistical analyses were performed with BlueSky Statistics software (version 7.10; BlueSky Statistics LLC, Chicago, IL), and *P*<0.05 was considered to be statistically significant for all analyses.

## Results

### Baseline Characteristics and Advanced HF Diagnosis

Of the 5321 patients in the MACHD Registry, 12 (0.2%) met the diagnostic criteria for advanced HF at the time of baseline assessment (prevalent advanced HF) and were excluded from subsequent analyses. Of the 5309 patients without advanced HF at baseline, 432 (8%) developed advanced HF during follow-up. Table 1 compares the baseline characteristics of the patients that developed advanced HF to patients that did not develop advanced HF. The patients that developed advanced HF group were older and more likely to have severe CHD, comorbidities, ventricular dysfunction, valvular dysfunction, cardiac implantable electronic devices, and higher model for end-stage liver disease excluding international normalized ratio score at the time of baseline assessment. Table 2 shows the congenital heart lesions. Table S2 shows the European Society of Cardiology criteria for advanced that were present in the 432 patients with advanced HF at the time of diagnosis.

**Table 1.**
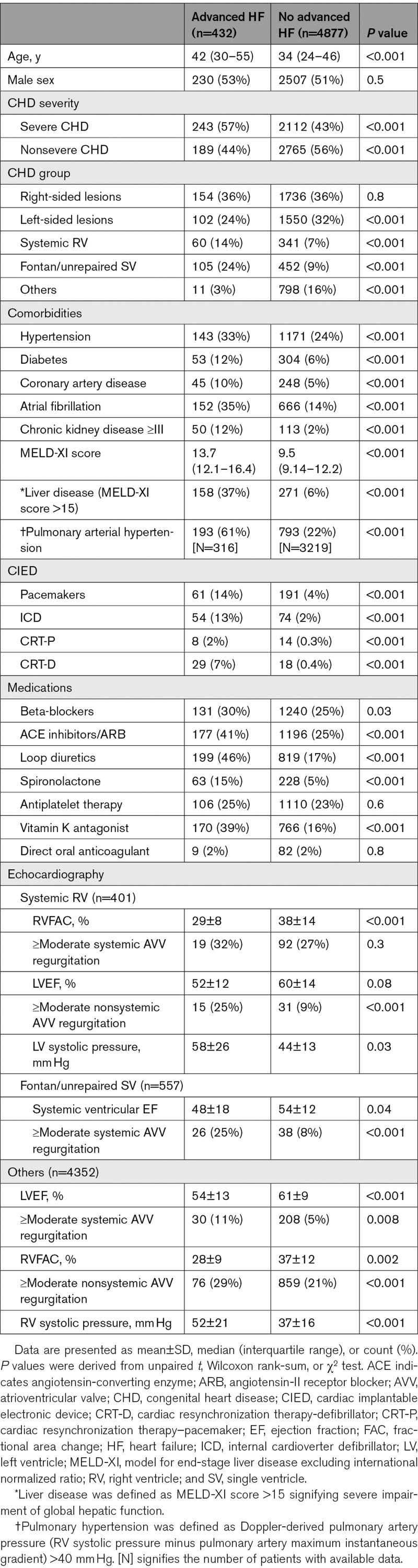
Baseline Clinical Indices (n=5309)

**Table 2.**
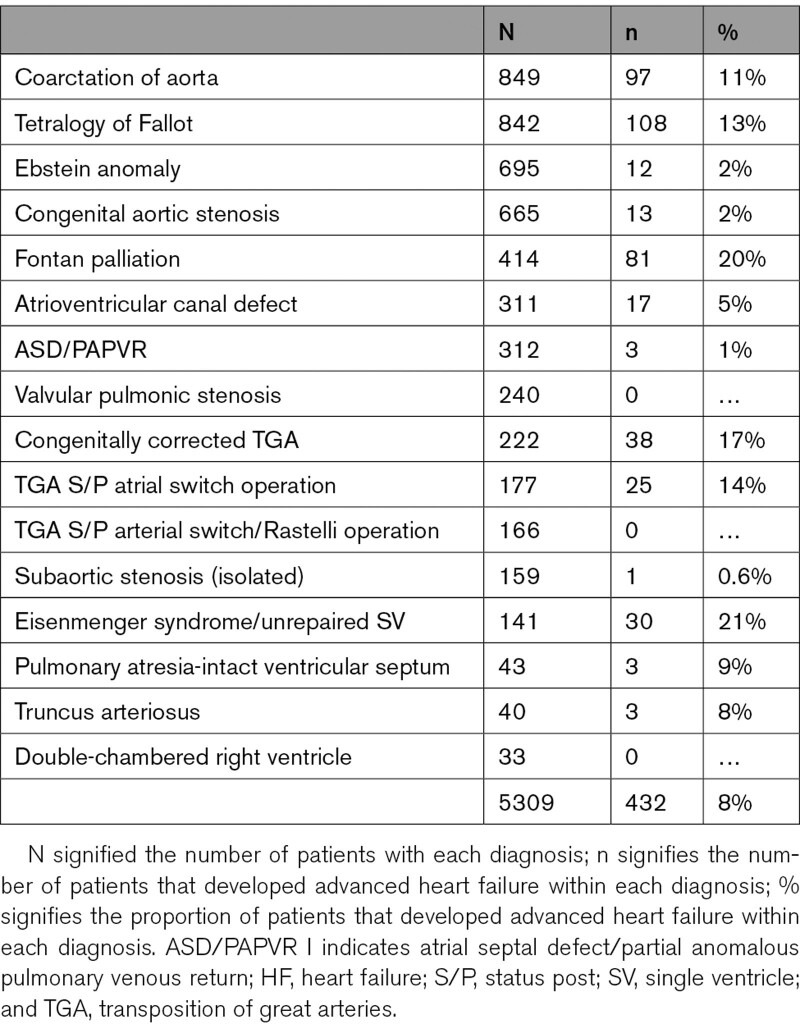
Congenital Heart Lesions of Patients With Advanced HF

**Figure 1.**
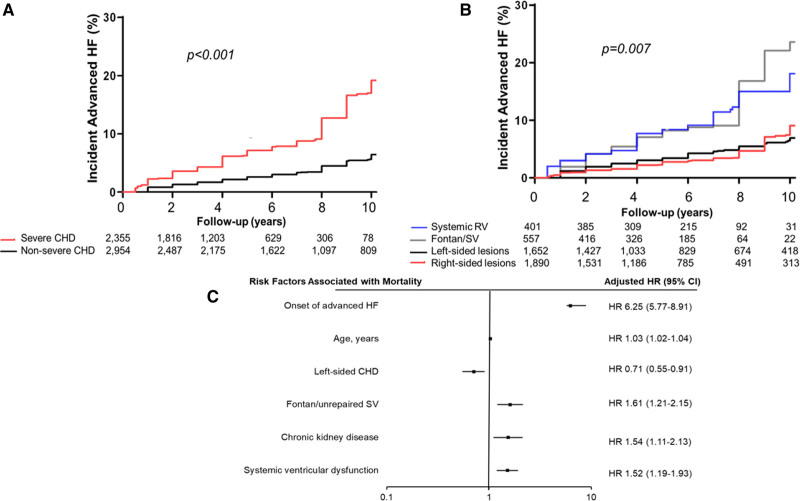
**Cumulative incidence of advanced heart failure (HF) stratified based on congenital heart disease (CHD) severity and anatomic/physiological classification. A** shows CHD severity, and **B** shows anatomic/physiological classification. The *P* values were derived from log-rank test comparing the different strata. Cox regression model showing risk factors associated with all-cause mortality among the 5309 patients that without advanced HF at the time baseline assessment (**C**). Only the covariates with statistically significant association (*P*<0.05) with all-cause mortality are shown. For the complete list of covariates in the Cox model, please see Table 4. HR indicates hazard ratio; RV, right ventricle; and SV, single ventricle.

The median interval from the baseline assessment to the diagnosis of advanced HF was 4.9 (1.1–7.3) years, and the 10-year cumulative incidence of advanced HF was 11% (95% CI, –14), corresponding to 1.1% per year. The 10-year cumulative incidence of advanced HF was higher in patients with severe CHD as compared to those with nonsevere CHD (19% versus 6%, *P*<0.001; Figure 1A), and severe CHD was associated with a 3-fold increase in the risk of advanced HF after adjustment for age, sex, and comorbidities (hazard ratio, 3.05 [95% CI, 2.49–3.74]; *P*<0.001; Table 3).

**Table 3.**
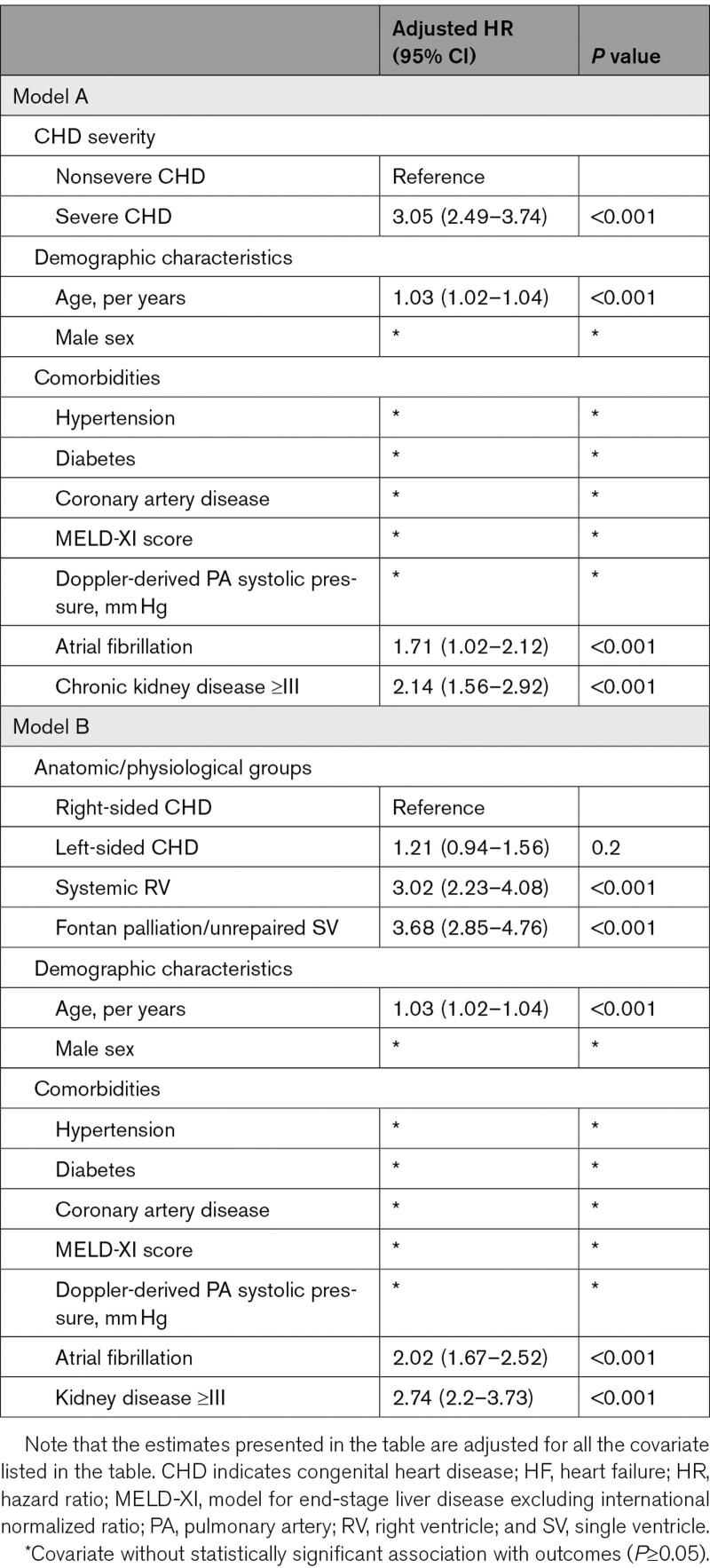
Multivariable Cox Model Showing Risk Factors Associated With Advanced HF

**Table 4.**
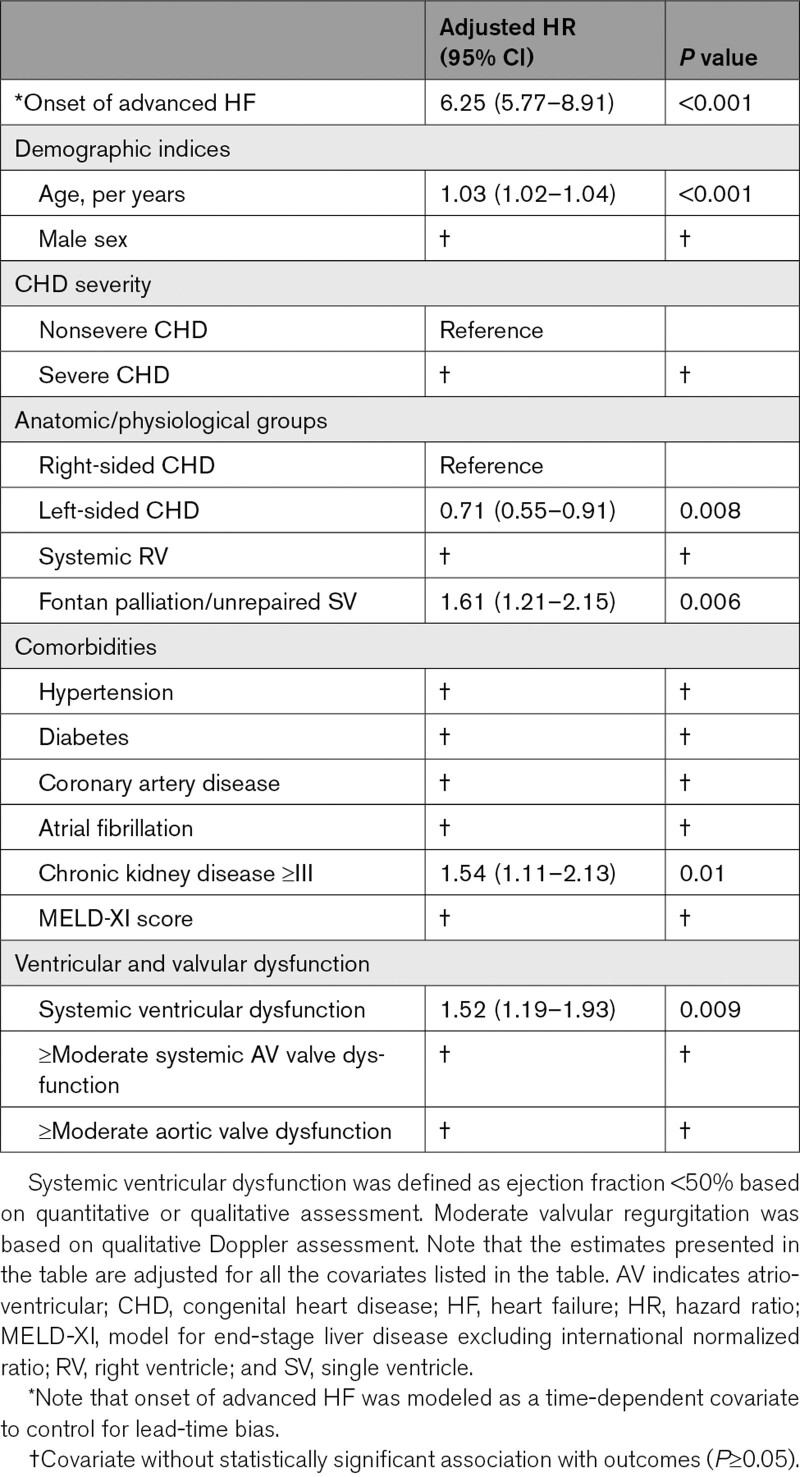
Multivariable Cox Regression Model Showing the Risk Factors Associated With All-Cause Mortality Among the 5309 Patients Without Advanced HF at Baseline Evaluation

Using the patients with right-sided lesions as the reference group, the 10-year cumulative incidence of advanced HF was similar in patients with left-sided lesions (7% versus 9%, *P*=0.8), but was higher in patients with systemic right ventricle (18% versus 9%, *P*<0.001), and patients with Fontan palliation/unrepaired single ventricle (24% versus 9%, *P*<0.001; Figure 1B). Both systemic right ventricle and Fontan palliation/unrepaired single ventricle were independently associated with higher risk of advanced HF after adjustment for age, sex, and comorbidities (Table 3).

### Advanced HF and All-Cause Mortality

The 5309 patients without advanced HF at baseline were followed for a median of 6.8 (2.3–10.8) years, and during this period 533 (10%) patients died. Development of advanced HF was associated with a 6-fold increase in all-cause mortality (hazard ratio, 6.25 [95% CI, 5.77–8.91]; *P*<0.001) after adjustment for age, sex, CHD severity, CHD lesion subgroup, comorbidities, and ventricular/valvular dysfunction (Figure 1C, Table 4).

### Clinical Outcomes in Patients With Advanced HF (n=432)

Of the 432 patients with advanced HF, 344 (80%) died during follow-up, and the median age at the time of death was 46 (33–51) years, and median interval from advanced HF diagnosis to death was 27 (16–41) months yielding an annual mortality rate of 38% per year. The annual mortality rate was significantly higher in the patients with advanced HF as compared to those without advanced HF (38% versus 0.9%, *P*<0.001).

Of the 432 patients with advanced HF, 152 (35%) had cardiac implantable electronic devices, of which 89 were defibrillators (implantable cardioverter defibrillator n=54 and cardiac resynchronization therapy with defibrillator n=29). Of the 89 patients with defibrillators, 61 (69%) devices were implanted for primary prevention, whereas 28 (31%) devices were implanted for secondary prevention. There were 24 appropriate shocks (6.6% per year) and 19 inappropriate shocks (5.3% per year). There was no significant difference in the annual incidence of appropriate shocks between the patients that received the device for primary prevention versus secondary prevention (5.9% versus 7.3% respectively, *P*=0.1).

**Figure 2.**
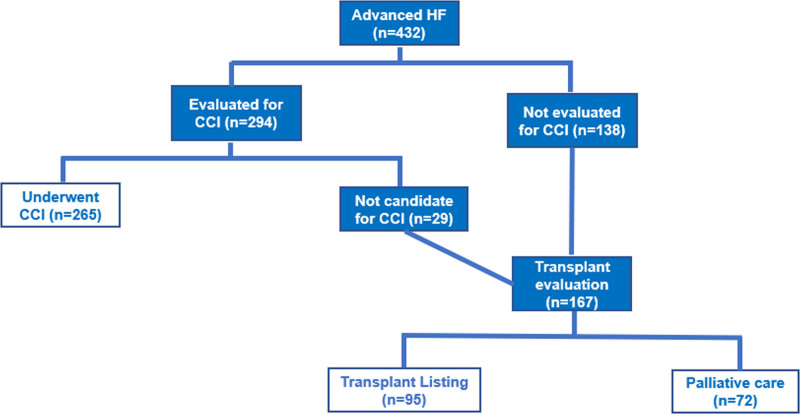
**Flowchart showing the different treatment pathways among the 432 patients with advanced heart failure (HF) during follow-up.** Of the 203 that were discharged home after conventional cardiac intervention (CCI), 62 patients were subsequently referred for transplant evaluation because of persistent or worsening HF symptoms, and all 62 patients were declined for transplant listing.

Figure 2 shows the treatment pathways and outcomes of the 432 patients that developed advanced HF. Of the 432 patients with advanced HF, 294 patients were evaluated for CCI, of which 265 underwent CCI, and the most common procedures were Fontan conversion operation (n=53), pulmonary valve replacement (n=82), tricuspid valve replacement (n=64), mitral valve replacement (n=61), aortic valve replacement (n=46), venous baffle revision (n=1), coronary artery bypass graft (n=39). The decision to proceed with CCI was based on the judgment of the primary cardiologist, and rationale was to delay heart transplant for as long as possible (n=151) or for palliation in patients that were not deemed suitable candidates for transplant (n=114). Of the patients that underwent CCI, 62 patients were subsequently referred for transplant evaluation because of persistent or worsening HF symptoms. All 62 patients were declined for transplant listing because they were considered to be high-risk candidates for transplant.

Seven patients (Fontan n=2, tetralogy of Fallot n=1, coarctation of aorta n=1, congenitally corrected transposition of aorta n=2, and complete transposition of aorta status post atrial switch operation n=1) received ventricular assist devices as destination therapy (HeartMate II n=4, HeartWare n=2, and total artificial heart n=1). Ventricular assist devices were implanted as destination therapy in 4 patients and as bridge to transplant in 3 patients. All 7 patients died during follow-up, and the cause of death was multi-system organ failure in 3 patients, early postoperative death following implantation of ventricular assist device in 2 patients, intracranial hemorrhage in 1 patient, and septic shock in 1 patient. The interval between the implantation of ventricular assist device and the time of death was 7 months (range 1 week to 4 years) for the patients that received the device as destination therapy, and 16 months (3 weeks to 19 months) for the patients that received the device as bridge to transplant.

Among the 167 patients referred for transplant evaluation, 95 (57%) were listed for transplant, whereas 72 (43%) were considered to have prohibitive risk for transplant, and hence received palliative care. Of the 95 patients listed for transplant, 43 (45%) patients were transplanted (isolated heart transplant n=41, and combined heart-liver transplant n=2), and 52 (55%) patients were not transplanted by the end of the study period because of death before transplant or because suitable donor allografts were not available. Of the 43 patients that underwent transplant, 8 (19%) patients died and the median interval from transplant to the time of death was 28 (1–71) months. The causes of death were early postoperative death following heart transplant (n=1), sepsis (n=5), stroke (n=1), and metastatic hepatocellular carcinoma after combined heart-liver transplant for Fontan-associated liver disease (n=1). The 1-year survival post-transplant was 91%.

**Figure 3.**
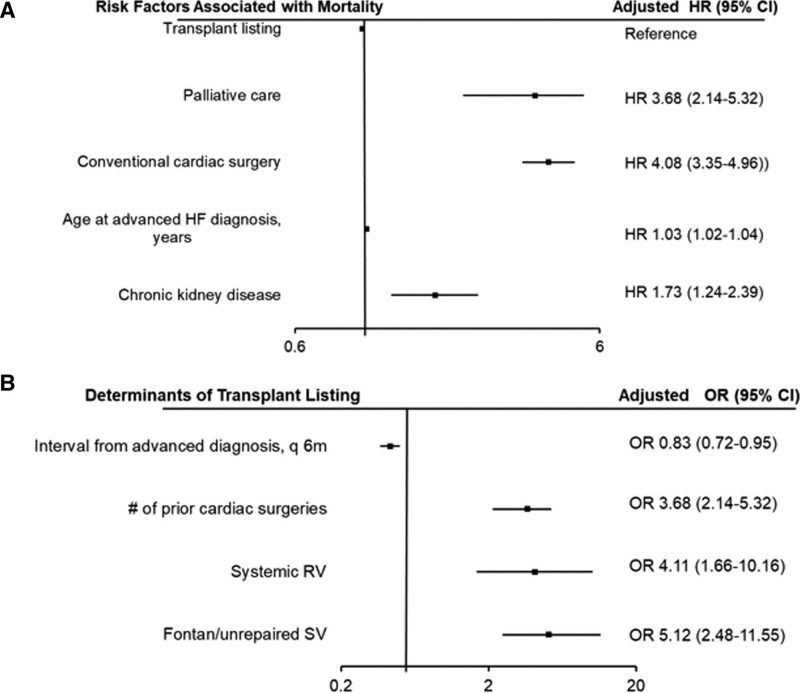
**Cox regression model showing the relationship between the different treatment pathways and all-cause mortality among the 432 patients with advanced heart failure (HF). A**, Logistic regression model showing the relationship between interval from advanced HF diagnosis (the interval from initial diagnosis of advanced HF to the initial presentation to the transplant clinic) and subsequent transplant listing among the 167 patients that underwent transplant evaluation (**B**). Only the covariates with statistically significant association (*P*<0.05) and outcome are shown. For the complete list of covariates in the Cox and logistic regression models, please see Table S4. HR indicates hazard ratio; OR, odds ratio; q6m, every 6 mo; RV, right ventricle; and SV, single ventricle.

Compared with the patients that underwent CCI, those that underwent transplant evaluation were older, more likely to have systemic right ventricle, and more likely to have Fontan palliation/unrepaired SV (Table S3). Using the transplant listing pathway as the reference group, CCI (hazard ratio 4.08 [95% CI, 3.35–4.96]) was associated with all-cause mortality after adjustment for age, sex, CHD severity, CHD lesions subgroup, and commodities (Figure 3A, Table 5). Furthermore, the interval between initial diagnosis of advanced HF and initial evaluation in the transplant was associated with lower odds of being listed for transplant (odds ratio, 0.83 [95% CI, 0.72–0.95]; *P*<0.001) after adjustment age, sex, CHD severity, CHD lesions subgroup, and commodities (Figure 3B, Table S4).

**Table 5.**
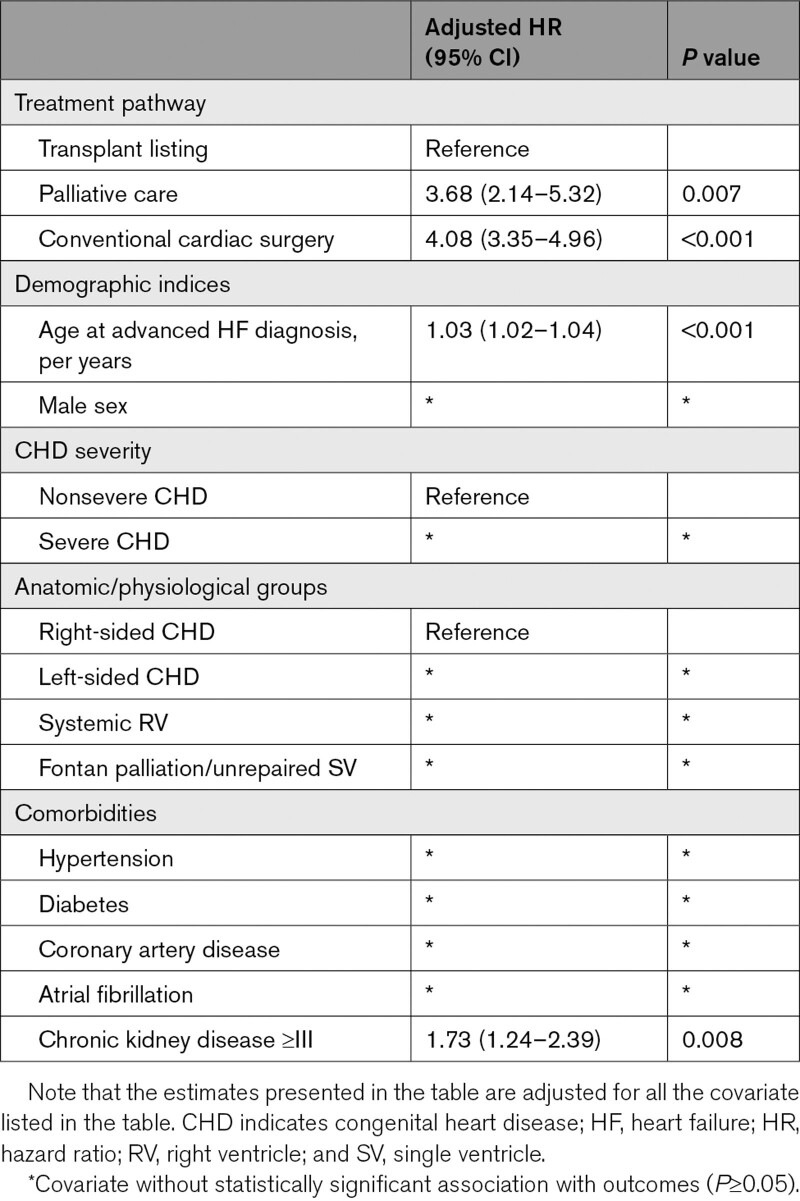
Multivariable Cox Regression Model Assessing the Relationship Between Treatment Pathway and All-Cause Mortality Among the 432 Patients With Advanced HF

## Discussion

In this study, we assessed the incidence and prognostic implications of advanced HF in adults with CHD, and the relationship between the different treatment strategies for advanced HF and all-cause mortality. The main finding are (1) the 10-year cumulative incidence of advanced HF was 11% corresponding to 1.7% per year, and new-onset advanced HF was more common in patients with severe CHD, systemic right ventricle and Fontan palliation/unrepaired single ventricle; (2) advanced HF was independently associated with all-cause mortality; (3) among the patients with new-onset advanced HF, CCI was associated with a higher risk of mortality as compared to transplant listing; (4) the interval between onset of advanced HF and initial evaluation in the transplant clinic was an important determinant of the likelihood of transplant listing and in turn, a determinant of all-cause mortality.

HF is an important determinant of hospitalization, healthcare resource utilization, and mortality in adults with CHD.^1–4,7,8^ There are many risk factors associated with HF, and interventions that reduce the burden of HF have been shown to improve survival in some CHD diagnoses.^11–15^ Although advanced HF (stage D HF) is a likely outcome for some adults with CHD, especially the patients with severe or complex CHD, there are limited data about this disease entity.^8,16,17^ The current study provides an estimate of the risk of developing advanced HF, and the risk factors associated with new-onset advanced HF such as CHD severity and complexity. More importantly, we observed that the onset of advanced HF was associated with 6-fold increase in the risk of mortality suggesting that the onset of advanced HF marks a critical point in the natural history of HF beyond which the risk of mortality rises exponentially. These dismal results are comparable to the report from Dunlay et al^6^ showing a median survival of 12 months and 49% mortality at 1 year after diagnosis of advanced HF in patients with acquired heart disease although their cohort had a median age of 77 years (3 decades older than the CHD cohort).

Most of the pathologies in patients with CHD are due to structural abnormalities, such as valvular/conduit dysfunction and baffle dysfunction, and as a result, CCI are often the first line treatment strategy offered to the patient.^12,18–20^ In this study, we observed that CCI was associated with a significantly higher risk of mortality as compared to being listed for transplant. However, a direct comparison cannot be made between survival after CCI versus transplant listing based on our data because of selection bias resulting from a nonrandomized assignment of therapy. One of the indications for recommending CCI was to delay heart transplant for as long as possible in patients with target lesions amenable to surgery. However, we observed that all the patients that had CCI before referral for transplant evaluation were subsequently rejected and that a longer interval from the initial onset of advanced HF to transplant evaluation was associated with lower odds of being listed for transplant. This suggests that an early identification of patients with advanced HF, and early referral for transplant evaluation would likely improve the odds of being listed, and in turn, improved the odds of survival since transplant listing is associated with a higher survival outcome. Furthermore, the median survival after implantation of ventricular assist device was <2 years, and this highlights the need to optimize the selection criteria for advanced HF therapy in this population. We also observed a high incidence of appropriate device shocks in patients that received implantable cardioverter defibrillator regardless of whether it was implanted for primary versus secondary prevention. This finding attests to the fact that patients with advanced HF represent a high-risk group, and further studies are required to determine whether changes in the criteria for implantable cardioverter defibrillator implantation in this population will improve outcomes.

Another important observation from this study was that chronic kidney disease was associated with all-cause mortality in the overall cohort, as well as within the subset of patients that developed advanced HF. This is consistent with previous studies that identified chronic kidney disease as a marker of adverse outcome in patients with cardiovascular disease.^21,22^ We postulate that this finding is due to the complex interaction between the heart and the kidneys, whereby cardiac dysfunction can lead to renal dysfunction (because of systemic venous congestion and renal hypoperfusion for low cardiac output), while renal dysfunction can lead to cardiovascular remodeling and dysfunction from chronic inflammation.^23–26^

### Study Strengths and Limitations

This is a retrospective study based on patients that were followed in a single referral center, and it is therefore prone to selection and ascertainment bias. Although we controlled for potential confounders using robust statistical methods, it is possible that the observed differences in mortality between the different treatment pathways might have been influenced by differences in the baseline characteristics of the patients since these therapies were not randomly assigned. For instance, all 62 patients that initially underwent CCI and subsequently referred for transplant evaluation were declined for transplant listing suggesting that the patients that underwent CCI may represent a sicker subset. These limitations can only be adequately controlled by conducting a randomized controlled trial. Additionally, we had limited data about all the factors that were considered (sensitization, psychosocial factors, and procedural risk associated with chest reentry resulting from collaterals and anatomic complexity) before deciding on transplant listing versus palliative care in the patients that underwent transplant evaluation. These factors will also introduce a selection bias for the different treatment pathways. Furthermore, we did not assess the impact of frailty on outcomes because data about fragility were not available in our cohort.

### Conclusions

The data presented in this study suggest that the onset of advanced HF marks a critical point in the natural history of HF beyond which the risk of mortality rises exponentially. Additionally, CCI was associated with significantly higher risk of mortality as compared to transplant listing, and the longer the interval from the initial onset of advanced HF to transplant evaluation, the lower the odds of the patient being listed for transplant. Therefore, early identification of patients with advanced HF, and a timely referral for transplant evaluation may offer the best chance of survival for these critically ill patients. The European College of Cardiology criteria for advanced (Table S1) are based on indices that are usually obtained during routine cardiac evaluation, and hence should be easy to apply in clinical practice. Further studies are required to determine whether the application of these data will improve outcomes in this population.

## Article Information

### Sources of Funding

Dr Egbe is supported by National Heart, Lung, and Blood Institute grants (R01 HL158517 and R01 HL160761). The MACHD (Mayo Adult Congenital Heart Disease) Registry is supported by the Al-Bahar Research grant.

### Disclosures

None.

### Supplemental Material

Tables S1–S4

## Supplementary Material


