# Supplementary material for: Incidence and Outcomes of Advanced Heart Failure in Adults With Congenital Heart Disease
Source: Circ Heart Fail. 2022 Oct 4;15(12):e009675. doi: 10.1161/CIRCHEARTFAILURE.122.009675 (PMC9760468; doi:10.1161/CIRCHEARTFAILURE.122.009675)
Supplement: Supplementary file 1 [file hhf-15-e009675-s001.pdf]

Supplementary Table S1: European Society of Cardiology Criteria for Advanced HF

**Criteria #1: Episodes of congestion, low output, or malignant arrhythmias**		
Congestion	Physical exam	Peripheral edema
	Physical exam, CXR	Pulmonary edema
Low output	Doppler, Fick, thermodilution	Cardiac index <2.5 l/min/m^2
Malignant arrhythmia	ECG, device report	Sustained VT, VF, ICD shock
**Criteria #2: Severe cardiac dysfn**		
LV (systemic) systolic dysfn	Echo, cardiac MRI	LVEF <40%
RV (non-systemic) systolic dysfn	Echo, cardiac MRI	RVFAC <35% or RVEF <50%
Severe LV (systemic) diastolic dysfn	Echo	Echo (all of the following)
		LVEF≥40
		LA volume index >34 ml/m^2
		Grade II/III diastolic dysfunction
		OR
	Cardiac catheterization	PAWP >15 mmHg
**Criteria #3: Severe exercise impairment**		
Impaired aerobic capacity	CPET	Peak VO_2 <50% of predicted
	6-MWT	<300 m
**Criteria #4: Severe and persistent symptoms of heart failure**		
NYHA IIIb or IV	Chart review	Symptoms of heart failure at rest or with minimal exertion

HF: heart failure; CXR: chest x-ray; ECG: electrocardiogram; VT: ventricular tachycardia; VF: ventricular fibrillation; ICD: implantable cardioverter defibrillator; MRI: magnetic resonance imaging; LV: left ventricle; RV: right ventricle; EF: ejection fraction; FAC: fractional area change; LA: left atrium; CPET: cardiopulmonary exercise test, 6-MWT: six-minute walk test; NYHA: New York Heart Association; PAWP: pulmonary artery wedge pressure; VO_2: oxygen consumption.

Note that in patients with systemic RV, the RV systolic function criteria were applied to the systemic ventricle

Note that in patients that underwent more than one type of test for the assessment of a specific criterion, the determination of whether a criterion was present (or absent) was based on a hierarchical selection of test as follows: Pulmonary congestion (physical exam > CXR); Low output (Fick > thermodilution > Doppler); Ventricular systolic function (cardiac MRI > echo); ventricular diastolic dysfunction (cardiac catheterization > echo); Exercise impairment (CPET > 6-MWT)

To have advanced HF, a patient must meet all 4 criteria, despite attempts to optimize medical, surgical, and device therapy. Note that definitions for the European Society of Cardiology Criteria for Advanced HF were modified to suit the congenital heart disease population

Supplementary Table S2: European Society of Cardiology Criteria for Advanced HF

	n with data	n (%) with positive criteria (%)
**Criteria #1: Congestion, low output, or malignant arrhythmias**		
Congestion (peripheral edema)	432	392 (91%)
Congestion (pulmonary edema)	432	365 (61%)
Low output	395	189 (48%)
Malignant arrhythmia	432	34 (8%)
**Criteria #2: Severe cardiac dysfunction**		
LV (systemic) systolic dysfunction	432	134 (31%)
RV (non-systemic) systolic dysfunction	366	289 (79%)
Severe LV (systemic) diastolic dysfunction	325	129 (40%)
**Criteria #3: Severe exercise impairment**		
Cardiopulmonary exercise test	417	405 (97%)
6-Minute walk test	261	223 (85%)
**Criteria #4: Severe and persistent symptoms of heart failure**		
NYHA IIIb or IV	432	432 (100%)

LV: left ventricle; RV: right ventricle; NYHA: New York Heart Association; HF: heart failure

To have advanced heart failure, a patient must meet all 4 criteria, despite attempts to optimize medical, surgical, and device therapy. Note that definitions for the European Society of Cardiology Criteria for Advanced HF were modified to suit the congenital heart disease population

Supplementary Table S3: Comparison of Baseline Characteristics Patients that Underwent Conventional Cardiac Intervention versus Transplant Evaluation

	CCI (n=265)	Transplant Evaluation (n=167)	p
Age, years	41 (30-54)	44 (32-57)	0.03
Male sex	144 (54%)	86 (52%)	0.6
**CHD severity**			
Severe CHD	154 (59%)	87 (52%)	0.2
Non-severe CHD	109 (41%)	80 (49%)	0.2
**CHD group**			
Right-sided lesions	94 (35%)	60 (36%)	0.8
Left sided lesions	68 (26%)	35 (21%)	0.3
Systemic RV	23 (7%)	37 (22%)	0.008
Fontan/unrepaired SV	74 (28%)	31 (19%)	0.04
Others	6 (2%)	4 (2%)	0.9
**Comorbidities**			
Hypertension	92 (35%)	51 (31%)	0.4
Diabetes	34 (13%)	19 (11%)	0.6
Coronary artery disease	22 (8%)	23 (14%)	0.07
Atrial fibrillation	98 (37%)	54 (32%)	0.3
Chronic kidney disease ≥III	31 (12%)	19 (11%)	0.9

CCI: conventional cardiac intervention; CHD: congenital heart disease; RV: right ventricle; SV: single ventricle. Note that Transplant evaluation group included patients that were subsequently listed transplant and those that subsequently received palliative care

Supplementary Table S4: Multivariable Logistic Regression Model Showing the Determinants of Transplant Listing (Being Accepted for transplant) Among the 167 patients Referred for Transplant Evaluation

	Adjusted OR (95% CI)	p
Interval from advanced HF, per 6 months	0.83 (0.72-0.95)	<0.001
**Demographic characteristics**		
Age, per years	---	---
Male sex	---	---
# of prior cardiac surgeries	0.96 (0.93-0.99)	0.03
**CHD severity**		
Non-severe CHD	Reference	
Severe CHD	---	---
**Anatomic/physiologic groups**		
Right-sided CHD	Reference	
Left-sided CHD	---	---
Systemic RV	4.11 (1.66-10.16)	0.002
Fontan palliation/unrepaired SV	5.12 (2.48-11.55)	<0.001
**Comorbidities**		
Hypertension	---	---
Diabetes	---	---
Coronary artery disease	---	---
Atrial fibrillation	---	--
Chronic kidney disease ≥III	---	---

CHD: congenital heart disease; RV: right ventricle; SV: single ventricle; OR: odds ratio; CI: confidence interval. --- signifies covariate without statistically significant association with outcomes ($p \geq 0.05$)

Note that the estimates presented in the table are adjusted for all the covariate listed in the Table.
